# IR-Drop-Based Temperature Distribution in Large-Size AMOLED Panel

**DOI:** 10.3390/mi15101236

**Published:** 2024-10-04

**Authors:** Qibin Feng, Hongtao Ren, Zhe Dong, Zi Wang, Guoqiang Lv

**Affiliations:** 1 Special Display and Imaging Technology Innovation Center of Anhui Province, National Engineering Laboratory of Special Display Technology, Academy of Opto-Electric Technology, Hefei University of Technology, Hefei 230009, China; fengqibin@hfut.edu.cn (Q.F.); wangzi@hfut.edu.cn (Z.W.); 2 School of Instrument Science and Opto-Electronics Engineering, Hefei University of Technology, Hefei 230009, China; 2022110019@mail.hfut.edu.cn (H.R.); dong395332023@163.com (Z.D.)

**Keywords:** AMOLED, IR-drop, temperature distribution, thermal simulation

## Abstract

Large-size and high-resolution AMOLED displays have become one of the most attractive display technologies. However, the dependence of the luminance of AMOLED on temperature severely limits wider applications. The accurate temperature distribution is important for implementing compensation into a panel to improve display uniformity. With the increase in size and resolution, the voltage drop (IR-drop) caused by the resistance of the power supply line cannot be ignored, which has influence on temperature distribution. Therefore, this paper proposes a temperature distribution analysis method based on IR-drop. Firstly, an accurate solution of IR-drop of AMOLED panels is achieved by exploiting the sparse representation in the field of artificial intelligence. Secondly, the IR-drop-based power model is established, and the output of the power model is used as the input of the AMOLED thermal simulation model. Finally, the temperature distribution of the AMOLED panel is obtained by finite-element analysis. The temperature measurements are performed on a 95-inch 8K AMOLED panel. The simulation results are compared with the actual measurements, and it is found that the temperature distribution based on IR-drop matches well with the actual measurements than that without considering IR-drop. The analysis method proposed in this paper presents high accuracy and high practicability.

## 1. Introduction

Active-matrix organic light-emitting diodes (AMOLEDs) have many advantages such as high dynamic range, fast response time and ultra-thinness [1,2]. In recent years, AMOLED displays have developed toward a large size, high resolution and better user experience. With the increase in size and resolution, IR-drop becomes inevitable. The IR-drop is the voltage drop along the power metal line from the driver at the panel edge to the pixel of an AMOLED panel, which leads to emission non-uniformity [3,4]. Meanwhile, because of the fact that most of the electrical power of an AMOLED panel is converted into heat, IR-drop will also cause temperature disturbance.

Although the brightness and luminous efficiency of AMOLEDs have achieved great improvement, their lifetime and stability are still important bottlenecks limiting their applications [5]. The illumination efficiency of an AMOLED heavily depends on temperature. The increased temperature of an AMOLED panel will cause three serious side effects: Firstly, it affects the uniformity of AMOLED panels [6]; secondly, it leads to changes in the crystallization or morphology of organic thin films [7,8,9]; and thirdly, it leads to black spots at short-circuit points inside the panels [10]. In order to improve the thermal stability of AMOLED panels, there exist two kinds of methods: One is to develop high-temperature-resistant organic materials, such as hole-transporting materials with high glass transition temperatures [11], and another is to establish efficient heat dissipation pathways. Boroumand, F.A. et al. found that, with the same driving voltage and input current density, temperature is directly proportional to the light-emitting area of AMOLEDs, and the main heat dissipation mechanism is thermal conduction between AMOLEDs and heat sinks [12]. No matter which method is used, it is necessary to achieve the precise temperature distribution of AMOLED panels to implement compensation. Zhang, W.W. et al. conducted a thermal simulation study of AMOLEDs using the finite-element method and pointed out that the surface properties of the substrate or cathode can significantly affect the temperature distribution of AMOLEDs [13]. Li, Y.T. et al. analyzed the temperature field and heat flow distribution of AMOLEDs using finite-element analysis software and found differences in the thermal effects of three commonly used encapsulation structures (traditional encapsulation structure of the back-cover type, hybrid encapsulation structure and Barix encapsulation structure) for AMOLED panels [14]. Lin, S.F. et al. established a temperature forecast model based on a neural network for different sections of an AMOLED panel [15]. However, none of the above studies considered the effect of IR-drop on temperature distribution.

In this paper, the analysis method based on IR-drop is proposed, which can help achieve the accurate temperature distribution of a large-size and high-resolution AMOLED panel. This paper is organized as follows: In Section 2, the IR-drop model based on sparse representation is presented, which can achieve accurate voltage in each pixel. In Section 3, the voltages are input into the AMOLED thermal simulation model to simulate temperature distribution. The IR-drop results and the temperature distribution results are introduced in Section 4. The actual temperatures are tested and the comparison between the simulated and experimental results are introduced in Section 5. Section 6 concludes this study.

## 2. Modeling IR-Drop of Large-Size and High-Resolution AMOLED

Because of the fact that most electric power used by AMOLEDs is converted into heat, the driving voltage of each pixel has a direct influence on temperature distribution. Existing IR-drop algorithms require very large amounts of computational power and a very long runtime for solving the IR-drop of a large-size, high-resolution AMOLED panel. In this section, an IR-drop algorithm based on sparse representation is proposed, which can help save computational resources to achieve a fast solution to IR-drop.

### 2.1. IR-Drop Model

**Figure 1:**
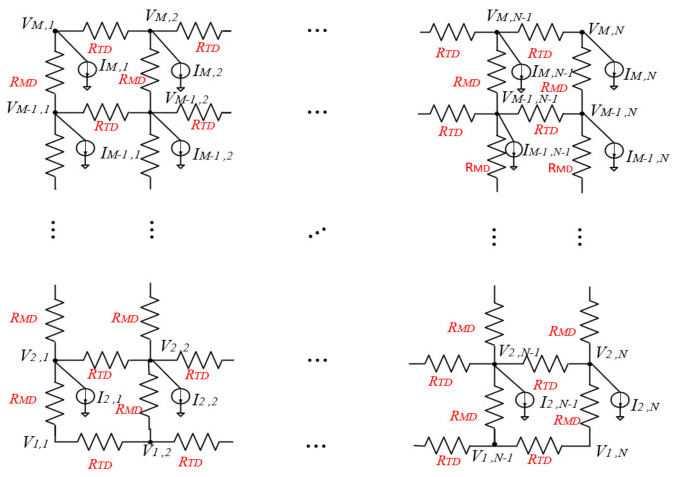
The equivalent circuit model of the driver circuit for the AMOLED panel.

In this paper, the equivalent circuit model of the driving circuit of an AMOLED panel with M×N resolution is built, as shown in Figure 1. The capacitance and inductance are usually ignored when performing static IR-drop analysis. At this point, the equivalent circuit model of the driving circuit of the AMOLED panel can be regarded as a purely resistive network, and this equivalent model includes only the equivalent AMOLED current source and the power supply metal line resistance.

As also shown in Figure 1, $R_{MD}$and$R_{TD}$ are the thin layer resistances of the power metal line segments between two adjacent sub-pixels along the latitude and longitude, respectively. $R_{MD}$and$R_{TD}$ can be obtained according to the following equations:

(1) $$R_{TD}=\rho \times \frac{l_{MD}}{h_{TD}{\times w}_{TD}}$$

(2) $$R_{MD}=\rho \times \frac{l_{TD}}{h_{MD}{\times w}_{MD}}$$

where $l_{TD}$ and $l_{MD}$ denote the lengths of the latitudinal and longitudinal power metal lines, respectively. $h_{TD}$ and $h_{MD}$ denote the line heights of the latitudinal and longitudinal power metal lines, respectively. $w_{TD}$ and$\;w_{MD}$ denote the line widths of the latitudinal and longitudinal power metal lines, respectively. ρ denotes the resistivity of the power metal lines.

According to the equivalent circuit theory, the equivalent circuit model of the driving circuit of the AMOLED panel shown in Figure 1 can be established as an IR-drop model represented by the vector matrix equation, as shown in (3).

(3) $$\left[\begin{array}{ccccc} {G^{\prime }}_{1,1} & C_{1} & 0 & \cdots & 0\\ C_{1} & {G^{\prime }}_{2,2} & C_{2} & \ddots & \vdots \\ 0 & C_{2} & \ddots & \ddots & 0\\ \vdots & \ddots & \ddots & {G^{\prime }}_{M-1,\;\;M-1} & C_{M-1}\\ 0 & \cdots & 0 & C_{M-1} & {G^{\prime }}_{M,M} \end{array}\right]\left[\begin{array}{c} V_{1}\\ V_{2}\\ \vdots \\ V_{M-1}\\ V_{M} \end{array}\right]=\left[\begin{array}{c} I_{1}\\ I_{2}\\ \vdots \\ I_{M-1}\\ I_{M} \end{array}\right]$$

where G is an (M×N)×(M×N) matrix that represents the conductance matrix for the interconnected resistors, V is an (M×N)×1 matrix that represents the vector matrix of all AMOLED node voltages, and I is an (M×N)×1 matrix that represents the vector matrix of all AMOLED node currents. C is an N×N matrix.

The sub-matrix ${G^{\prime }}_{i,i}$ represents the connectivity in the same row, and the value on the diagonal is the self-conductance of each node (i,j) in row i. The sub-diagonal value is the mutual conductance, which is negative. The sub-matrix $C_{i}$ represents the connectivity of two neighboring rows, the non-zero elements of the sub-matrix $C_{i}$ are all located on its diagonal, and its value is the conductance of the two neighboring rows, which is negative.

### 2.2. Sparse Representation of the IR-Drop Model

With the increase in resolution and size, the IR-drop model of an AMOLED has a high matrix order and fewer non-zero elements, which requires a larger running memory and longer running time for the existing algorithms. By exploiting the sparse representation in the field of artificial intelligence, only the non-zero elements in the conductivity matrix *G* are stored, which can help reduce the size of the matrix and make it possible to accurately solve the IR-drop of AMOLED panels with a large size and high resolution. Take the first column as an example, as shown in the following equation.

### 2.3. IR-Drop Model Solution

The AMOLED panel is supplied from a single edge, and the supply voltage is noted as $V_{dd}$. Thus, we can reformulate the circuit model by replacing the value of$V_{dd}$ by the voltage of all the power nodes and ignoring the Kirchhoff’s current law (KCL) equations for the power nodes.

(4) $${G^{\prime }}_{1,1}=\left[\begin{array}{ccc} 1 & & \\ & \ddots & \\ & & 1 \end{array}\right]$$

(5) $$I_{2}=\left[\begin{array}{c} V_{dd\;}*\frac{1}{R_{MD}}-I_{2,1}\\ \vdots \\ V_{dd\;}*\frac{1}{R_{MD}}-I_{2,N} \end{array}\right]$$

The mutual conductances between the power nodes and neighboring nodes are set to 0.

**Figure 2:**
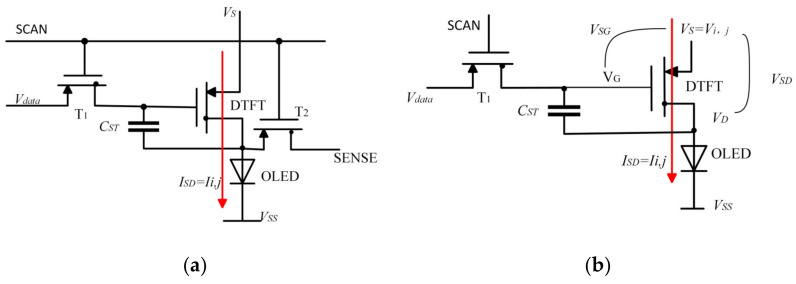
AMOLED pixel driver circuit. (**a**) 2T1C; (**b**) 3T1C.

**Figure 3:**
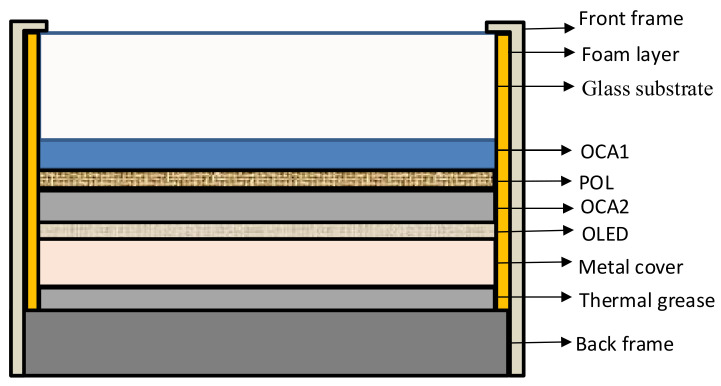
Structural diagram of the AMOLED panel.

In order to solve V and I in the IR-drop model, the exact I–V relationship has to be firstly determined. Figure 2 presents two kinds of driving circuits for AMOLEDs. It can be seen that no matter whether 2T1C (Figure 2a) or 3T1C (Figure 2b) is employed, the luminance of an OLED pixel is determined by the driving current $I_{SD}$ that is controlled by the driving thin-film transistor (DTFT). Therefore, I–V relationship of the DTFT is used for the IR-drop analysis, as shown as follows [16,17]:

(6) $$I_{i,j}=I_{SD}=\frac{1}{2}\mu_{n}C_{ox}\frac{W}{L}{\left(V_{SG}-|V_{th-DTFT}\;|\right)}^{2}\;\left(1+\lambda V_{SD}\right)$$

where $\mu_{n},C_{ox}$ and $\frac{W}{L}$ are the mobility, oxide capacitance and aspect ratio of the driving TFT, respectively. $\mu_{n}C_{ox}\frac{W}{L}$ = 200 $\frac{\mu A}{V^{2}}$. $V_{th-DTFT}$ is $V_{th}$ of the DTFT, and λ is the channel length modulation coefficient. $V_{SG}$ and $V_{SD}$ are the source-to-gate voltage and source-to-drain voltage, respectively.

$I_{i,j}$ and $V_{i,j}$ can be obtained by iteratively solving (3). The conductance matrix G of (3) is a positive definite matrix. If the iterations converge, the solution for $V^{k+1}$ should be closer to the solution of the original equation than for $V^{k}$, so a more accurate value of the node voltages can be obtained by replacing $V^{k}$ with $V^{k+1}$. The continuous iterations are designed herein to stop as the difference between two consecutives V’s through the iterations becomes smaller than the iteration error. The iteration result can be used as the real node voltage value for the IR-drop analysis, as shown in the following:

(7) $$\delta_{i,j}=V_{dd\;}-{\;V}_{i,j}$$

where $\delta_{i,j}$ is the IR-drop from $V_{dd}$ at node (i,j).

## 3. Temperature Distribution Analysis of AMOLED Panel

### 3.1. IR-Drop-Based Power Model

After obtaining the IR-drop at each node of the AMOLED panel, this paper further proposes an IR-drop-based power model. The output of the power model is used as the input to the AMOLED thermal simulation model in order to analyze the temperature distribution [18]. The energy conversion efficiency is set to η%, which means that (1−η)% of the electrical power is converted to heat. The IR-drop-based power model can be expressed using (8)–(10):

(8) $$P_{OLED\left(i,j\right)}=V_{OLED\left(i,j\right)}*I_{OLED\left(i,j\right)}*(1-\eta )\%$$

(9) $$V_{OLED\left(i,j\right)}=V_{i,j}-V_{SS}-V_{SD}$$

(10) $$I_{OLED(i,j)}=I_{i,j}$$

where $P_{OLED(i,j)}$ is the thermal input power of the node (i, j); $V_{OLED(i,j)}$ is the voltage of the node (i, j); and $I_{OLED(i,j)}$ is the current flowing through the node (i, j).

### 3.2. AMOLED Thermal Simulation Model

In this paper, a finite-element analysis is used to analyze AMOLED temperature distribution. As Figure 3 shows, the panel model consists of the glass substrate (0.7 mm), the OCA1 layer (0.1 mm), the POL layer (0.02 mm), the OCA2 layer (0.1 mm), the OLED layer (0.1 mm), the metal cover (0.15 mm), the thermal grease layer (0.08 mm) and the back frame (2.5 mm). The OLED layer consists of the organic light-emitting layer, the thin-film circuit layer and the silicon oxide insulation layer.

The material parameters of the model are listed in Table 1, and it is assumed that the thermal conductivity of all the materials does not vary with temperature.

## 4. Simulation Results

### 4.1. Results of IR-Drop

**Figure 4:**
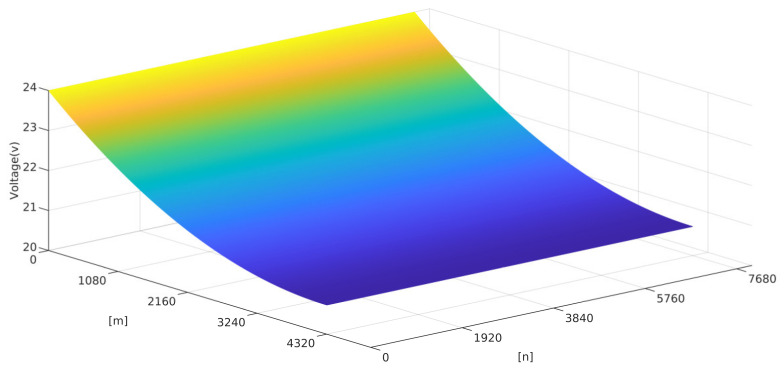
Results of $V_{i,j}$ distribution in OLED panel with 8 K resolution.

IR-drop analysis was performed for a 95-inch 8 K (4320 × 7680) AMOLED panel. $V_{dd}$ on the driver side of the panel is set to 24 V. Both ${\;R}_{TD}andR_{MD}$ in the IR-drop model are set to 0.5 Ω. The distribution of $V_{i,j}$ for the OLED panel with a resolution of 8K at a gray level of 255 is shown in Figure 4.

The commonly used IR-drop solutions, including conjugate gradient algorithm (CG), incomplete Cholesky conjugate gradient algorithm (ICCG) and block conjugate gradient algorithm (BCG), are performed for comparison with the proposed algorithm [19]. Table 2 presents the running times of various algorithms for solving the IR-drop of a large-size and high-resolution AMOLED panel. For the solution of 32 M nodes (8 K resolution), CG algorithm, ICCG algorithm and BCG algorithm runtimes were 10,173.64 s, 3379.66 s and 3635.39 s, respectively. The IR-drop algorithm with sparse representation only takes 120.72 s to achieve the exact solution, which means the proposed algorithm requires fewer computational resources than existing algorithms. The proposed IR-drop algorithm presents high practicability for large-size and high-resolution AMOLED panels.

### 4.2. Results of Temperature Distribution Simulation of AMOLED Panel

**Figure 5:**
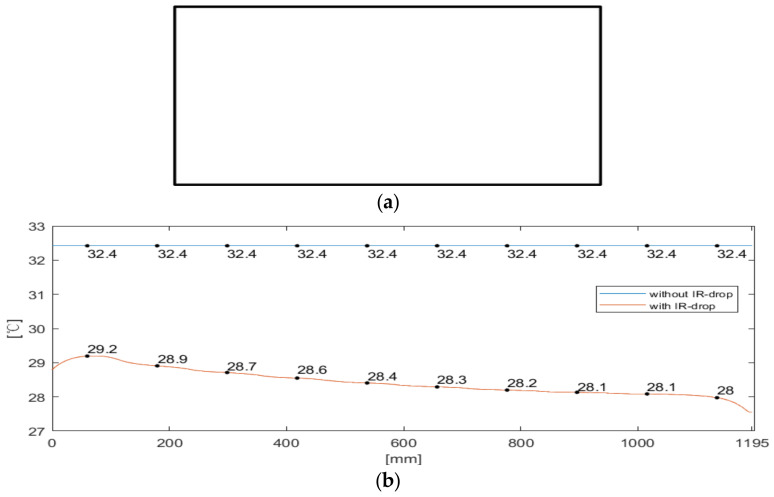
(**a**) All-white field image; (**b**) Temperature distribution simulation profile with IR-drop and without IR-drop of Case 1.

To sufficiently verify the proposed method, three different cases are simulated, including different images (all-white and checkerboard), different power, etc., as shown in Table 3. In Case 1, the test image is an all-white image, the driving power is 24 V × 11 A. In Case 2 and 3, the test image is the same of checkerboard image, but the driving power is defined as 24 V × 14 A for Case 2 and 24 V × 22 A for Case 3. The surface emissivity of the glass is set to 0.8, the surface emissivity of the ABS is set to 0.9, and the natural convection heat transfer coefficient of the glass is set to 1 $W\cdot m^{-2}\cdot K^{-1}$. In order to better validate the method proposed in this paper, the temperature distribution without IR-drop is also simulated. Figure 5a shows the all-white image, and Figure 5b shows the temperature distribution simulation profile with and without IR-drop.

**Figure 6:**
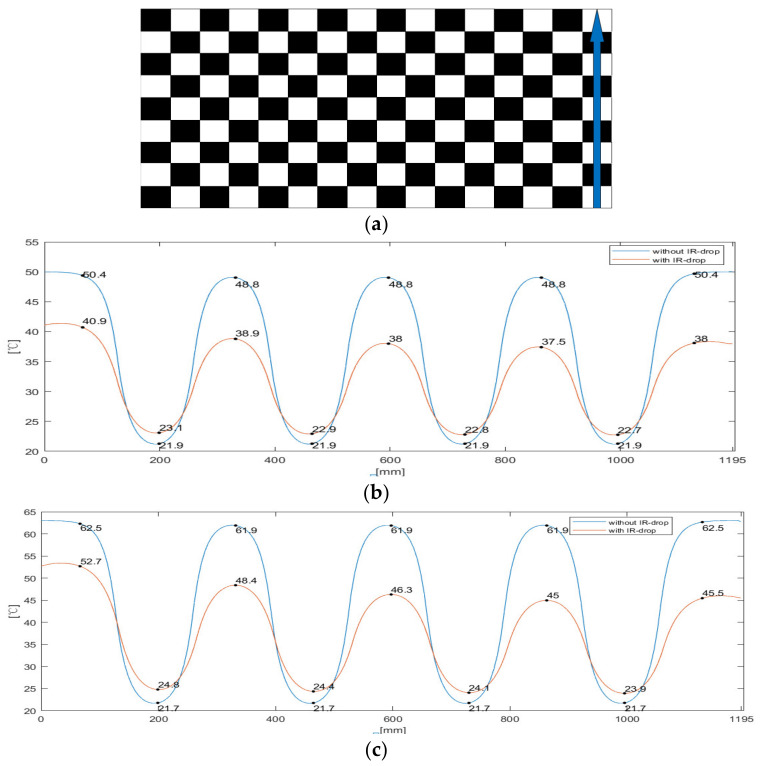
(**a**) Checkerboard image and a path for temperature simulation; (**b**) Case 2 temperature distribution simulation profile with IR-drop and without IR-drop; (**c**) Case 3 temperature distribution simulation profile with IR-drop and without IR-drop.

Checkerboard image contains all-white and all-black blocks and is usually used for fast and accurate evaluation of optical and thermal performance. We simulated the temperature distribution of an AMOLED that displayed a checkerboard image. The simulated results are shown in Figure 6. Figure 6a shows a checkerboard image and a path for temperature simulation, and Figure 6b,c show the simulated temperature profiles along the path in Figure 6a with and without IR-drop for Case 2 and Case 3, respectively.

## 5. Experimental Results and Discussion

**Figure 7:**
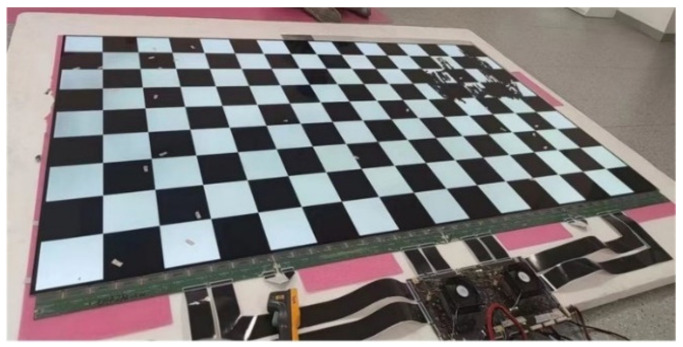
Physical view of the 95-inch 8 K-resolution AMOLED panel.

**Figure 8:**
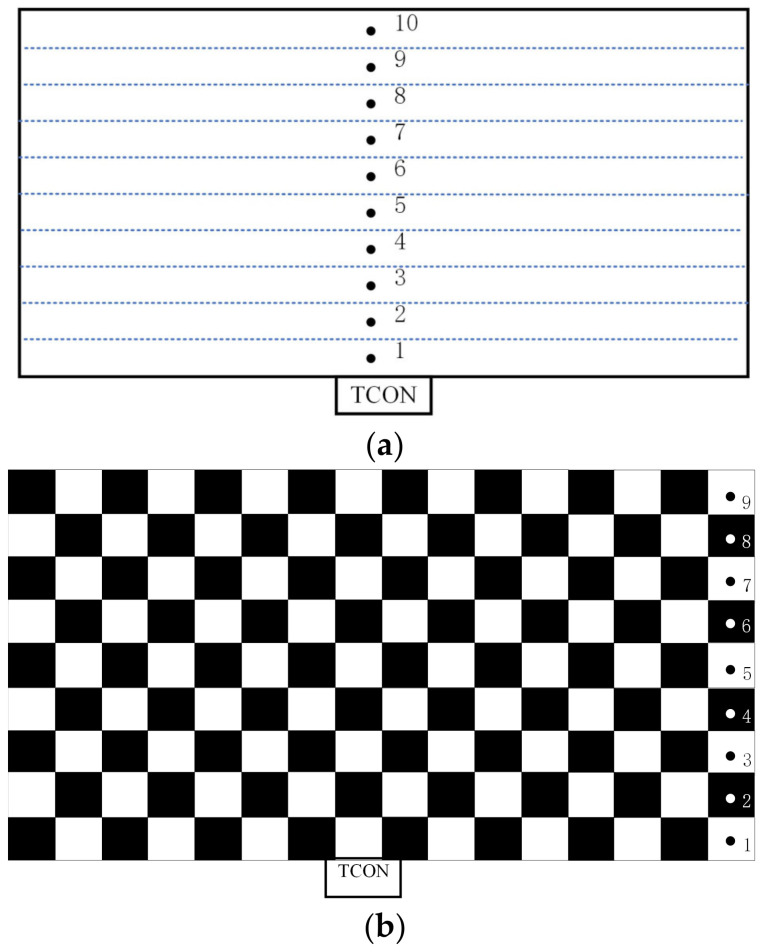
Distribution of measurement points. (**a**) All-white image; (**b**) Checkerboard image.

The temperature distributions of the three simulation cases were measured on a 95-inch 8K-resolution AMOLED panel. The measurement system is shown in Figure 7. The 95-inch 8K AMOLED panel with 7680 × 4320 pixels consists of more than 33 million organic light-emitting diodes. And the AMOLED panel has 40 ports connected to the driving board via flexible printed circuits (FPCs). IR-drop causes voltage differences at different locations on the panel, which affects the display performance, which affects the display performance. The temperatures were measured in a room-temperature environment of 19.8 °C. After lighting up the panel for 30 min, the temperatures of 10 measurement points for the all-white image and 9 measurement points for the checkerboard image, as illustrated in Figure 8, were recorded. The temperatures were measured by a platinum resistance thermometer with an accuracy of 0.1 °C.

**Figure 9:**
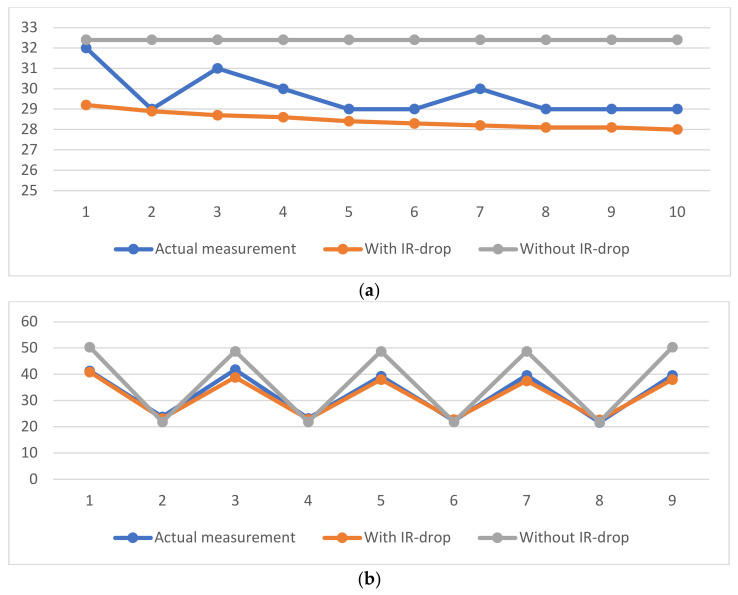

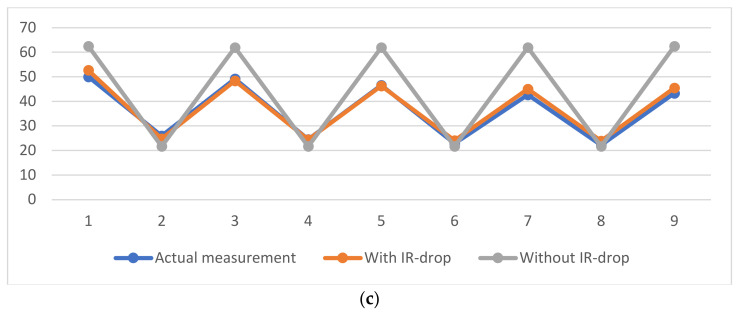
Temperatures of measurement points. (**a**) Case 1; (**b**) Case 2; (**c**) Case 3.

The actual and simulated temperatures are shown in Figure 9. The error analysis between the actual and simulated temperatures are shown in Table 4. In the simulated temperatures with IR-drop of Case 1, the simulated temperatures based on IR-drop decreases gradually with the increase in the distance between the measurement point and the driver side of the panel, which matches well with the actual temperatures. In the simulated temperatures without IR-drop, the temperatures remain constant with the position. At the 10th point, the errors between the two simulation temperatures and the actual temperature are 1 °C and 3.4 °C, respectively. In Case 2, the temperatures of both the white block and the black block in the IR-drop-based simulation decrease gradually with the increase in the distance between the measurement point and the driver side of the panel. The average simulated temperature in the white block is 42.1 °C and the average simulated temperature in the black block is 22.1 °C, with an average temperature difference of 20 °C, which match well with the actual temperatures. In the simulated temperatures without IR-drop, the temperatures of both the white block and the black block remains constant with the position. At the ninth point, the errors between the two simulated temperatures and the actual temperature are 1.6 °C and 10.8 °C, respectively. In Case 3, the temperatures of both the white block and the black block in the IR-drop-based simulation decrease gradually with the increase in the distance between the measurement point and the driver side of the panel. The average simulated temperature in the white block is 46.3 °C and the average simulated temperature in the black block is 22.5 °C, with an average temperature difference of 23.9 °C, which match well with the actual temperatures. In the simulated temperatures without IR-drop, the temperatures of both the white block and the black block remain constant, which present no difference with the different measurement positions. At the ninth point, the errors between the two simulated temperatures and the actual temperature are 2.2 °C and 18.6 °C, respectively. The comparison results show that the temperature distribution of the AMOLED panel based on IR-drop presents high accuracy.

## 6. Conclusions

This paper proposes a temperature distribution analysis method based on IR-drop, which enhances the accuracy of simulated temperature distribution for large-size and high-resolution AMOLED panels. The temperatures of the simulation match well with the actual measurements, which may help significantly improve the effectiveness of thermal evaluation for AMOLED panels and implement effective compensation.

## Figures and Tables

**Table 1:** Parameters of the material.

Structure	Material	Thermal Conductivity (W/m·K)
Glass substrate	Glass	1.1
OCA1	Olefin film	0.24
POL	Polymer film	0.2
OCA2	Olefin film	0.24
OLED	Nichrome film	43.8
Metal cover	Stainless steel	26.3
Thermal grease	Aluminum	1
Back frame	ABS	0.24
Front frame	ABS	0.24
Bumper	EPE	0.04

**Table 2:** Running times of different IR-drop algorithms.

Method	Time (s)
CG	10,173.64
ICCG	3379.66
BCG	3635.39
Proposed algorithm	120.72

**Table 3:** Thermal simulation cases.

No	Cases	Driving power (Voltage× Current)
1	All-white image	24 V×11 A
2	Checkerboard image	24 V×14 A
3	Checkerboard image	24 V×22 A

**Table 4:** Error analysis between actual and simulated temperatures.

Temperature of the Test Points (°C)		1	2	3	4	5	6	7	8	9	10
Case 1	Actual measurement	32	29	31	30	29	29	30	29	29	29
	With IR-drop	29.2	28.9	28.7	28.6	28.4	28.3	28.2	28.1	28.1	28
	Error	2.8	0.1	2.3	1.4	0.6	0.7	1.8	0.9	0.9	1
	Without IR-drop	32.4	32.4	32.4	32.4	32.4	32.4	32.4	32.4	32.4	32.4
	Error	0.4	3.4	1.4	2.4	3.4	3.4	2.4	3.4	3.4	3.4
Case 2	Actual measurement	41.3	23.8	41.8	23.2	39.3	22.3	39.6	21.7	39.6	/
	With IR-drop	40.9	23.1	38.9	22.9	38	22.8	37.5	22.7	38	/
	Error	0.4	0.7	2.9	0.3	1.3	0.5	2.1	1	1.6	/
	Without IR-drop	50.4	21.9	48.8	21.9	48.8	21.9	48.8	21.9	50.4	/
	Error	9.1	1.9	7	1.3	9.5	0.4	9.2	0.2	10.8	/
Case 3	Actual measurement	50	26	49.2	24.5	46.6	22.8	42.7	22.2	43.3	/
	With IR-drop	52.7	24.8	48.4	24.4	46.3	24.1	45	23.9	45.5	/
	Error	2.7	1.2	0.8	0.1	0.3	1.3	2.3	1.7	2.2	/
	Without IR-drop	62.5	21.7	61.9	21.7	61.9	21.7	61.9	21.7	62.5	/
	Error	11.9	2.6	11.5	1.1	14.1	0.6	18	1.2	18.6	/

## Data Availability

The data that support the findings of this study are available from the corresponding authors upon reasonable request.
